# Mr. Dunning's Cases of Spina Bifida

**Published:** 1806-03

**Authors:** R. Dunning

**Affiliations:** Plymouth Dock


### *To the Editors of the Medical and Physical Journal.*

GENTLEMEN,

ON January 17, 1806, I delivered Mrs. P. of a very fine boy. I remarked, however, with the greatest concern, in the site of the lowest lumbar vertebra, a large oblong aperture, whose greatest diameter was at least an inch and a half. The external integuments terminated suddenly at the edge of it; nothing indeed can be more entire than this large chasm. At somewhat more I thought than the depth of half an inch was seen, apparently, the spinal marrow in its envelope; but instead of an opake white, it was in no inconsiderable degree of a reddish colour; I say, apparently, because I did not minutely examine it, lest I should too much attract the attention of the poor mother, at a moment, when their solicitude with respect to the state of the child is always extreme; the babe in every other respect was perfect, and seemed to be strong and well. I sent to the nurse a piece of Bayntun's adhesive plaster to be applied over the aperture; on the following morning the space between the plaster and what I have said appeared to be the medulla spinalis, was entirely filled up, and the plaster a little protruding. In the mean time the child had been restless, but we did not think it ill; the next day, from the moisture underneath it, the plaster had slid off. I found the incipient tumor had now considerably increased, that a membranous expansion on the surface of it had cracked seemingly in several places, and that a little of an aqueous discharge had exuded. The child had taken the breast and been fed, and its evacuations had been natural; yet it was evidently on the decline, and I ascertained at this time that he had not the least power over the lower extremities, which were entirely pendulous; and the nurse assured me too, that he had never stretched out nor drawn them up.

At the end of a week the tumour had attained the size and pretty much the shape of a middle-sized turnip; having ing remained nearly stationary for three or four days it began rapidly to collapse and sphacelate, and the child died on the fifteenth day.

The medical world will hardly, I believe, give me credit for detailing this case of spina bifida as a novelty, although its progress has been a little more marked perhaps than it often has been. The following occurrence will at once explain my motives.

Two years ago, I removed from a child a few days after its birth, a firm fleshy tumour, nearly the size of a pullet's egg, which appended by a peduncle an inch long from the middle and hinder part of the neck; the peduncle was somewhat larger than the largest goose-quill; I passed through it a needle with a double ligature, and tied it on each side; in passing the needle I observed with much surprize a single drop of water to escape; Mr. Little, who was with me, and who perfectly coincided with me in his opinion, with respect both to the propriety and mode of removing this præternatural excrescence, remarked it also with equal surprize.

The day after the application of the ligature the babe became restless, and a good deal disordered; the tumor rapidly sphacelated of course; the attending circumstances hourly grew worse, and having lain a few days under a train of symptoms which usually mark cerebral affections, the child died.

No surgeon, I have the great satisfaction fully to believe, would have shrunk a moment from removing this pendulous tumour in the way that I did; certainly there were no characters either in the appearance or feel of it, that could possibly suggest even the most remote suspicion that it was at all connected with the vertebræ. I may add too that the child was extremely fat; a circumstance, as fat is generally largely apportioned to this part, which would have rendered any connection with the spine much less discoverable had any suspicion of it occurred at the time. But from the circumstances which arose, I was afraid then, and am more afraid now, that the peduncle was in a small degree tubular, that it was connected with a small aperture in the cervical vertebra, that the drop of fluid which escaped was similar to that discharged in the preceding case, and that the fatal event happened in both instances from the same train of causes.

A similar case may again present itself, and similar means may without hesitation be again resorted to for its removal; I feel it therefore a professional duty to place

 this this event on record; and I do not know where I can give it a more conspicuous situation than in your widely circulating Journal.

From the continued friction to which this excrescence would be necessarily exposed, and much more from the connection, if it existed, between it and the medulla spinalis, there is unquestionably the greatest reason to conclude, that it would have ulcerated at no distant period. It is hardly necessary to add, that whenever this happened the event would have been the same. Be that however as it might, I do not anticipate that any opprobrium will attach to me from the publication of this case; and truly, if there does, I shall not feel it. It will hardly be out of place to remark, that the back is the frequent seat of various tumours.

I was favoured a few days since with an opportunity of seeing Mr. Veitch, surgeon in the Royal Naval Hospital here, remove an immense fatty mass from the back of a seaman. It was seated on the middle of the back, and lay perfectly superficial under the integuments on the fascia of the muscles, to which it was but slightly connected by cellular membrane and several nutrient vessels, which tho' small, were much disposed to bleed. This mass was of more than twenty years growth; in its circumference more than two feet, and weighed 4 lb. 2 oz.

If I were at all conscious that my approbation would give Mr. Veitch pleasure, or in any shape serve him, I should say with great gratification, that no surgeon in the world I believe could have dissected out this tumour with more neatness or expedition.

---

As I continue to believe that early and repeated rupturing the vaccine vesicle renders vaccination in certain constitutions, what I have ventured to term *incomplete*, and is therefore among the most frequent causes of those failures that occur in the practice. As I have insisted at considerable length on this circumstance; have published several facts and observations of my own; and many others with which I have been favoured by very respectable practitioners, all tending strongly to confirm the truth of this conjecture; and as these have failed to engage that attention, which it appears to me the subject deserves, I request you will do me the favour to print the following extract from the Minutes of the original Vaccine-Pock Institution. The case case it details is entirely in accord with the facts and observations I have adduced; coming from high authority, it will probably ensure more notice than many similar ones either from my neighbours or myself. Dr. Wood, in his valuable paper in the last Journal, seems to think that two vesicles are always necessary; surely then we may justly suspect, that one ruptured vesicle may be, now and then, in certain constitutions insufficient.

Tuesday, January 7, 1806, Dr. Nelson communicated that he had inoculated with vaccine matter the child of Mr. Josse, in Vigo Lane, Carnaby Market, about three years and a half ago. The child had only *one vesicle, from which matter was taken to inoculate two others,* of whom one went through the cow-pox in the usual way; the other had an irregular disease, no vesicle being produced, though the punctured part was surrounded by a large areola.

It appears that Mr. Josse's child had the small-pox in the month of November last, about a fortnight after having been exposed to the contagion of that disease. There is now perceptible *an irregular cicatrix* from vaccination.

I am, &c.

*Plymouth Dock, Feb.* 5, 1806. R. DUNNING.

